# Unveiling the dynamics of the breast milk microbiome: impact of lactation stage and gestational age

**DOI:** 10.1186/s12967-023-04656-9

**Published:** 2023-11-06

**Authors:** Parul Singh, Noora Al Mohannadi, Selvasankar Murugesan, Fajr Almarzooqi, Basirudeen Syed Ahamed Kabeer, Alexandra Katharina Marr, Tomoshige Kino, Tobias Brummaier, Annalisa Terranegra, Rose McGready, François Nosten, Damien Chaussabel, Souhaila Al Khodor

**Affiliations:** 1 College of Health and Life Sciences, Hamad Bin Khalifa University, Doha, Qatar; 2 Research Department, Sidra Medicine, Doha, Qatar; 3 Shoklo Malaria Research Unit, Mahidol-Oxford Tropical Medicine Research Unit, Faculty of Tropical Medicine, Mahidol University, Mae Sot, Thailand; 4 Centre for Tropical Medicine and Global Health, Nuffield Department of Medicine, University of Oxford, Oxford, UK; 5 The Jackson Laboratories, Farmington, CT USA

**Keywords:** Breast milk, Microbiome, Preterm birth, Breastfeeding, Prematurity

## Abstract

**Background:**

Breast milk (BM) provides complete nutrition for infants for the first six months of life and is essential for the development of the newborn’s immature immune and digestive systems. While BM was conventionally believed to be sterile, recent advanced high throughput technologies have unveiled the presence of diverse microbial communities in BM. These insights into the BM microbiota have mainly originated from uncomplicated pregnancies, possibly not reflecting the circumstances of mothers with pregnancy complications like preterm birth (PTB).

**Methods:**

In this article, we investigated the BM microbial communities in mothers with preterm deliveries (before 37 weeks of gestation). We compared these samples with BM samples from healthy term pregnancies across different lactation stages (colostrum, transitional and mature milk) using 16S rRNA gene sequencing.

**Results:**

Our analysis revealed that the microbial communities became increasingly diverse and compositionally distinct as the BM matured. Specifically, mature BM samples were significantly enriched in *Veillonella* and *lactobacillus* (Kruskal Wallis; *p* < 0.001) compared to colostrum. The comparison of term and preterm BM samples showed that the community structure was significantly different between the two groups (Bray Curtis and unweighted unifrac dissimilarity; *p* < 0.001). Preterm BM samples exhibited increased species richness with significantly higher abundance of *Staphylococcus haemolyticus, Propionibacterium acnes, unclassified Corynebacterium species*. Whereas term samples were enriched in *Staphylococcus epidermidis, unclassified OD1,* and *unclassified Veillonella* among others.

**Conclusion:**

Our study underscores the significant influence of pregnancy-related complications, such as preterm birth (before 37 weeks of gestation), on the composition and diversity of BM microbiota. Given the established significance of the maternal microbiome in shaping child health outcomes, this investigation paves the way for identifying modifiable factors that could optimize the composition of BM microbiota, thereby promoting maternal and infant health.

**Supplementary Information:**

The online version contains supplementary material available at 10.1186/s12967-023-04656-9.

## Introduction

Breast milk (BM) is the first food for newborn infants and is recommended by the World Health Organization as the “exclusive diet” for the first 6 months of life [1, 2]. BM contains a unique and optimal combination of nutrients and bioactive components, including immunoglobulins and cytokines, bioactive lipids, human milk oligosaccharides (HMOs), microRNAs, hormones, and microorganisms, among others [3]. This unique composition of BM adapts to the need of the offspring and exhibits variations that extend across individuals, lactational stages, daily fluctuations, and even between feeding sessions [4]. The concentrations of these diverse components are also contingent upon factors such as diet, maternal genetic makeup, gestational age, and the health status of the mother [3].

It was once believed that the BM microbes were a form of extrinsic contamination and that human BM was a nearly sterile fluid, however this theory has now been rejected [5]. To date, many studies have concluded that BM is home to its own unique microbiome, including beneficial, commensal, and potentially probiotic bacteria [6–8]. The intricate and ever-evolving process through which the BM microbiota is introduced remains a subject of complexity, with facets yet to be fully understood.

Two conceivable mechanisms have been proposed to elucidate the introduction of milk microbiota. Firstly, the notion of "retrograde transfer" involves the external influx of bacteria, sourced from the areola skin and the oral cavity of the infant. The second mechanism, known as the "entero-mammary pathway," encompasses the migration of bacterial species emanating from the maternal gut to the mammary glands [3, 9].

The application of culture-independent molecular techniques, and particularly those based on 16S rRNA genes, allowed a complementary biodiversity assessment of the human milk microbiome [10]. Pioneering studies indicated a high complexity and inter- individual variability in the milk microbial communities with few genera (*Streptococcus*, *Staphylococcus*, *Propionibacterium*, *Corynebacteria*, *Pseudomonas*, *Ralstonia*, *Serratia*, *Sphingomonas*, and *Bradyrhizobiaceae*) representing approximately half of the bacterial community abundance [11]. Nonetheless, the relative proportional representation of these genera exhibited substantial variations across different subjects [11]. Other studies such as the MAMI study and CHILD cohort study also identified that the BM microbiota composition is diverse and mostly dominated by the “core genera” including *Staphylococcus* and *Streptococcus* species [12, 13].

The content of BM undergoes dynamic shifts during nursing to cater to the evolving needs of the developing newborn across various stages [14]. Around mid- pregnancy, the synthesis of colostrum commences and extends for approximately five days postpartum, followed by a gradual transition to transitional BM, which persists for around two weeks [14]. By the fourth week after childbirth, BM is fully maturate, maintaining relatively consistent composition throughout the remainder of the lactation period [14]. Previous studies have reported changes in BM microbiota over the course of lactation, for example colostrum samples were dominated by *Weissella*, *Leuconostoc*, *Staphylococcus*, *Streptococcus*, and *Lactococcus* [15]. In contrast, at one and six month postpartum, BM samples were enriched by representatives of the oral cavity such as *Veillonella*, *Leptotrichia*, and *Prevotella* [15].

Our current understanding of the BM microbiome predominantly stems from studies involving mothers with healthy pregnancies, a perspective that may not readily extend to mothers that develop pregnancy complications like preterm birth (PTB). Notably, components of BM beyond the microbiome (e.g., macronutrients, bioactives etc.) differ between mothers with uncomplicated pregnancies and those facing pregnancy-related complications [16, 17]. Given this, it is reasonable to assume similar disparities could manifest within the BM microbial communities.

Currently, only few studies have invesigated the BM microbiota in PTB [18–20]; however, most of these studies have limitations as highlighted in a recent publication by Asbury et al. [21]. Furthermore, these investigations have primarily concentrated on dissecting the microbial composition of preterm BM samples without comparing them to term birth controls. Thus, it is important to include a matched case–control cohort of women with term and preterm BM samples and study if pregnancy related complications can result in dysbiosis of BM microbiome and potentially impact the colonization of the infant gut microbiome and the developmental trajectory of their immune system.

As discussed earlier, several factors can influence the composition of the BM microbiota [3, 22]. Previous studies such as INSPIRE have shown that BM microbiota vary among cohorts originating from different geographical regions [6]. The landscape of advanced research often tilts towards high-income nations; thus, an empirical void emerges concerning investigations delving into the characterization of BM microbiota among mothers residing within resource-constrained contexts. This need is more prominent within Asian and marginalized refugee and migrant populations, wherein pregnancy-related complications carry profound implications for both maternal and infant well-being.

As part of our efforts to assess the molecular signature in pregnancy in mothers residing in low resources settings, we designed the MSP study [23, 24] with an aim to characterize cross-omic trajectories in pregnant women with and without pregnancy-associated complications to improve our understanding of their role in maternal and neonatal outcomes. Thus longitudinal, high frequency sampling was conducted as the part of the study to characterize microbial composition in various anatomical sites in pregnant women including BM samples collected postpartum [23, 24]. This is the first study to be conducted to characterize the BM microbiome in Karen and Burman women [23, 25].

We hypothesize considerable differences in the composition of preterm and term BM samples. Since the vast majority of the neonates in our study population were exclusively breastfed, we anticipate this is as the important source of infant gut colonization. The impact of pregnancy related complications on the mother’s milk microbiota could translate to changes in the infant gut microbial colonization and long-term health outcomes of preterm infants, especially considering the high rates of morbidity and lack of resources in this vulnerable population.

## Materials and methods

### Study participants and sample size

The Shoklo Malaria Research Unit (SMRU), a field station of the Faculty of Tropical Medicine at Mahidol University (Bangkok, Thailand), which is a part of the Mahidol-Oxford Research Unit, invited women with unremarkable medical and obstetric histories to participate [23]. The majority of this nomadic population live in modest, rural settlements. To create the cohort from this low-resource context, women with singleton viable pregnancy were enrolled in the molecular signatures in pregnancy (MSP study) between 2016 and July 2018 (n = 381) [23]. The presented study is a nested case–control study carried out at Sidra Medicine with a subset of samples selected from the MSP study participants as follows: 18 women delivering preterm and 30 matched controls (without pregnancy associated complications, who delivered at term (≥ 37 weeks), the case–control matching was done based on age, parity, and gravida. The MSP study has received the ethical approval from the Institutional Review Board (IRB) of Sidra Medicine under (IRB protocol #1,705,010,909), by the ethics committee of the faculty of Tropical Medicine, Mahidol University, Thailand (TMEC 15–062), the University of Oxford Central University Research, UK (OxTREC: 33–15), Trial registration number NCT02797327. The study was conducted in full conformity with the Declaration of Helsinki and followed regulations of the ICH Guidelines for Good Clinical Practice.

### Sample collection

At each collection, two BM samples were collected from each woman with PTB (n = 18), and matching controls (n = 30). Since it is possible that some maternal areolar skin microbiota will be sampled during BM collection; to provide an accurate representation BM microbiota two samples were collected from each women:

- One ‘clean sample’ was collected using an aseptic technique from the left breast
- One ‘natural sample’ was collected from the right breast

Each clean and natural sample were collected at three time points:

- 0–3 days postpartum (colostrum)
- 7–15 days postpartum (transitional milk)
- 2 months postpartum (mature milk)

Mothers were instructed to express the BM manually, only the left breast was cleaned with a povidone cotton swab before collection (clean, breast was thoroughly cleaned with water after the procedure), whereas the sample from the right breast was collected without cleaning (natural). Once expressed the first three drops were discarded and approximately 3 ml were collected in sterile falcon tubes, transferred to cryotubes tubes, and stored at − 80 °C till further processing.

### Microbial DNA extraction from BM samples

Approximately 2 ml of BM sample was centrifuged at 13,000 *g*, 4  °C, 20 min to pellet the microbial cells. DNA was extracted using the QIAamp Fast DNA Stool Mini Kit using a modified protocol. Briefly pelleted cells were resuspended in 600 µl of InhibitEX buffer, further homogenization was performed by vortexing with 0.2 g of sterile zirconia/silica beads (diameter, 0.1 mm; Biospec Product, ROTH, Karlsruhe, Germany), and incubation at 70 °C for 10 min to finish the lysis. The supernatant (600 mL) was transferred into a 2.0 mL microcentrifuge tube containing 25 mL Proteinase K. The subsequent steps were carried out as per the instructions of the QIAamp DNA stool MiniKit. The eluted DNA samples (50 µl) were stored at − 20  C until library preparation.

### Bacterial 16S rRNA PCR amplification and high throughput sequencing

Polymerase chain reaction (PCR) was used to amplify the 16S rRNA variable regions V1 and V3 using the amplicon primers with adapters (underlined)Forward:

5′TCGTCGGCAGCGTCAGATGTGTATAAGAGACAGAGRGTTTGATCMTGGCTCAG’3

Reverse: 5’GTCTCGTGGGCTCGGAGATGTGTATAAGAGACAGGTNTTACNGCGGCKGCTG’3 Illumina MiSeq 16S Metagenomic Sequencing Library Preparation protocol (http://support.illumina.com/downloads/16s_metagenomic_sequencing_library_preparation.html) was used to for amplicon library preparation. Samples were multiplexed using the Nextera XT Index kit (Illumina, San Diego, USA) according to the manufacturer’s instructions. Illumina MiSeq platform (Illumina, San Diego, USA), at the Sidra research facility was used for sequencing of the final pooled product using a MiSeq Reagent Kit v3 (paired end 2 × 300 bp).

### 16S sequence data processing and statistical analysis

Fast QC [http://www.bioinformatics.babraham.ac.uk/projects/fastqc] was used to assess the sequencing quality. Quantitative Insights into Microbial Ecology (QIIME2; version 2019.4.0) software package [26, 27] was used to input the demultiplexed sequencing data. Samples with less than sampling depth and were excluded from the final analysis. The data were denoised with DADA2 [28]. The data was then imported into R (RStudio v 2022.2.3.492 with R v 4.0.5) [29] for further evaluation. Observed OTUs, Chao1 [30], Shannon [31], Simpson [32], Pileous evenness indices [33], faith’s phylogenetic diversity (PD) [34], were used to quantify alpha diversity. Individual diversity measures can reflect many different aspects of diversity might be influenced by different assumptions; thus, each metric can be interpreted slightly differently. For instance, faith's phylogenetic diversity (faith’s PD) is a phylogeny-based metrics with an assumption that sample may have some number of highly related organisms (same genus or same phyla) is not as diverse as a sample comprised of organisms with greater phylogenetic distances (for different phyla or different genus). ACE and Chao are an indicator of species richness (total number of species in a sample) that is sensitive to rare OTUs (singletons and doubletons), Shannon and Simpson are an indicator of species evenness (proportional distribution of the number of each species in a sample). The alpha diversity metrics were measured and reported simultaneously. Weighted Unifrac, Unweighted Unifrac [35], Bray–Curtis, and Jaccard distance metrics [36] were used to measure beta diversity. UniFrac distance metric differ from other dissimilarity measures such as Bray–Curtis and Jaccard in that it incorporates information on phylogenetic distances between observed taxa. The beta diversity metrics were measured, the significant ones were reported. The Adonis was employed to establish significance, and PCoA was utilized as an ordination approach. Taxonomic classification was performed utilizing the Greengenes database [37], any sequences that were unassigned or archaeal, unclassified bacteria, mitochondria and chloroplasts were filtered out from the downstream analysis using filter_pollution and tidy_dataset functions of R package “MicroEco” [38]. We used Wilcoxon or Kruskal–Wallis nonparametric statistical tests, and Benjamini-Hochberg (BH) correction was used to compute the false discovery rate (FDR), with a *p*-value of 0.05 considered significant for all tests.

## Results

### Description of study subjects

Clinical, demographic, and pregnancy outcome characteristics data of women included in this study are summarized in Table 1. BM samples were analyzed from a subset of 48 women (18 women who experienced preterm birth and 30 age-matched women who experienced term birth). At the time of enrollment into the cohort, there were no significant differences in maternal height, weight, body mass index or delivery mode between preterm and term groups (Table 1). The mean gestational age at delivery for those who delivered preterm, and term was 36.2 and 39.5 weeks respectively. Apart from one participant in the term group, all the study participants included had normal vaginal delivery. Overall, 61.1% of mothers delivering preterm used antibiotics (at any time during their pregnancy) compared with 16.6% of term deliveries, with significant differences were observed at the time of delivery (*p* = *0.036*). As anticipated, infants born prematurely exhibited reduced birth weight and head circumference compared to the term controls (Table 1).

**Table 1:** Clinical parameters of the study cohort

	Term Birth (TB, n = 30)	Preterm Birth (PTB, n = 18)	p-values
Age at conception in years; Median (IQR)	23.5 (21–26.8)	21.5 (20–24.5)	0.3628
Height at conception in cm; Median (IQR)	151 (149–153)	154 (150–156)	0.136
Weight at conception in kilograms: Median (IQR)	48 (43–55)	48 (42.2–48.9)	0.3696
BMI at conception; Median (IQR)	20.9 (19.4–23.5)	20.1 (18.2–20.4)	0.1506
Outcome EGA (days); Median (IQR)	280 (270–284)	254 (242–255)	**9.277E-09**
Infant birth weight in Kg: Median (IQR)	3.07 (2.96–3.3)	2.26 (1.98–2.44)	**6.893E-08**
Infant Head circumfrence in cm: Median (IQR)	33 (32.4–33.6)	31 (30–31.5)	**2.219E-07**
BMI Categories n (%):			
Under weight	5 (27.5)	4 (13.3)	0.2145
NORMAL weight	11 (61)	17 (56.6)	0.7624
Over weight	1 (5.5)	7 (23.3)	0.1096
Obese	1 (5.5)	2 (6.6)	0.8776
Delivery (%):			
Vaginal	29 (96.6)	18 (100)	0.2731
Caesarean section	1 (3.3)	0 (0.0)	0.8776
Maternal Ethnic Group (%):			0.1587
Karen	22 (73)	9 (50)	0.1018
Burmese	8 (26.6)	8 (50)	0.2059
Gravida (%):			
1	11 (36.6)	8 (44.4)	0.5937
2	9 (30)	8 (44.4)	0.3111
3	5 (16.6)	1 (5.5)	0.2598
≥ 4	5 (16.6)	1 (5.5)	0.2598
Parity (%):			
0	12 (40)	9 (50)	0.499
1	10 (33.3)	8 (44.4)	0.4414
2	5 (16.6)	0 (0)	0.06725
≥ 3	3 (10)	1 (5.5)	0.5896
Maternal Antibiotic Exposure (%):	5 (16.6)	11 (61.1)	**0.004891**
1st trimester pregnancy	0 (0)	1 (5.5)	0.192
2nd trimester pregnancy	1 (3.3)	0 (0)	0.4337
3rd trimester pregnancy	1 (3.3)	2 (1.1)	0.2812
Delivery	5 (16.6)	8 (44.4)	**0.03603**

The significant p-values are in bold

The p-values where calculated using Wilcox and chi-square test (R.4.3.1 version)

### Overall sequencing results

Overall, 268 samples were sequenced including negative control (no-template amplification), after removal of samples with low read count (5 samples including the negative control) a total of 263 samples with an average sequencing depth of 20,212 ± 18,163 reads remained. The features that had a count of less than 10 were filtered out leaving 6900 features or amplicon sequence variants (ASVs). Finally, the feature table was rarefied to 5000 sequences per sample (Additional file 1: Fig. S1), the rarefaction curve tapered with increasing sequencing depth suggesting that the microbial population was sufficiently represented. After rarefying, 28 phyla, 465 genera and 645 species were taxonomically assigned.

### Clean and natural breastmilk samples show similar taxonomic diversity and composition

**Fig. 1:**
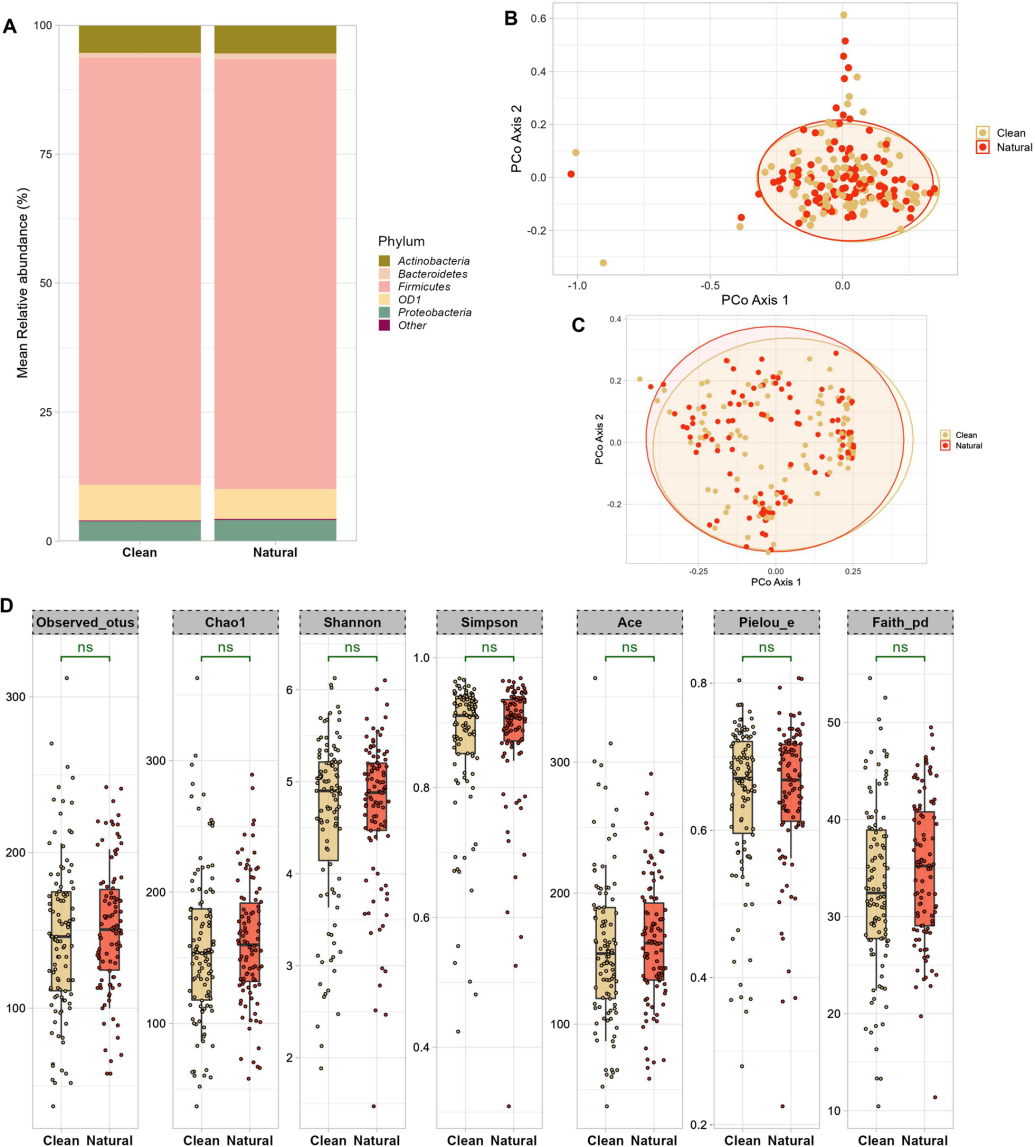
Comparison of the microbiota composition and diversity in Clean and Natural BM samples. (**A**) Relative abundances of the top five most abundant bacterial phyla in the two groups (**B**) PCoA plots showing the beta diversity measure using weighted unifrac distances (*p* = 0.73) and (**C**) Bray Curtis distance (*p* = 0.978); *p* values were determined by ADONIS; gold: clean, orange: natural. (**D**) Boxplots of Alpha-diversity indices: Observed OTUs; Chao1; Shannon; Simpson; Ace; Pielou_e; Faith_pd. Boxes represent the interquartile range (IQR) between the first and third quartiles (25th and 75th percentiles, respectively), and the horizontal line inside the box defines the median. Whiskers represent the lowest and highest values within 1.5 times the IQR from the first and third quartiles, respectively. Statistical significance was identified by the Wilcoxon test with false discovery rate (FDR)-Benjamini-Hochberg (BH) corrected *p* values. ns = non-significant; gold: clean, orange: natural. The figure was generated using (RStudio v 2022.2.3.492 with R v 4.0.5)

Some species and genera commonly detected from human milk, such as *Corynebacterium acnes* and *Staphylococcus epidermidis*, are also inhabitants of the human skin [39]. A previous study postulated that skin bacteria present on the surfaces of the nipple or areola could potentially access the mammary glands via ducts during breastfeeding [40]. As a result, when collecting BM samples for microbiota analysis, careful consideration must be given to the potential for skin contamination. To mitigate this, we employed two sampling approaches: natural collection (without aseptic application) and clean collection (preceded by gentle cleansing using a povidone cotton swab). In terms of microbial composition, the dominant phylum was *Firmicutes* (83%), followed by OD1 (candidate phylum *Parcubacteria*, 6.31%), *Actinobacteria* (5.45%), *Proteobacteria* (3.96%), and *Bacteroidetes* (1%) (Fig. 1A). At genus level *Streptococcus* (46.8%) and *Staphylococcus* (23.3%) and were the top two genera followed but others such as *unclassified OD1*, *Veillonella, Corynebacterium, Propionibacterium, Lactobacillus *etc*.* (Additional file 1: Fig. S2A). The profile of clean and natural samples was similar at phylum, genus and species levels (Fig. 1A, Additional file 1: Fig. S2A/B), no significant differences were found in the abundance of any taxa at all levels between the two groups of samples.

Beta diversity analysis indicates the extent of similarities and differences among microbial communities [36]. To quantify beta diversity, both non-phylogenetic (Bray–Curtis’s dissimilarity) and phylogenic methods (Unifrac distance) were used (Fig. 1B, C). We found no significant differences between the clean and natural BM samples in Bray–Curtis (*Pseudo-F* = 0.531, *p* = 0.98, ADONIS) and when using the weighted UniFrac distance (*Pseudo-F* = 0.55, *p* = 0.748, ADONIS). Alpha diversity metrics summarize the structure of an ecological community with respect to its richness (number of taxonomic groups), evenness (distribution of abundances of the groups), or both [41]. We applied both non-phylogenetic and phylogenetic alpha diversity indices, including observed OTUs (*p* adj = 0.66, Wilcox test), Faith’s PD (*p* adj = 0.66, Wilcox test), Shannon’s index (*p* adj = 0.96, Wilcox test), Pielou’s evenness (*p* adj = 0.96, Wilcox test), Simpson’s index (*p* adj = 0.98, Wilcox test), Chao1 (*p* adj = 0.66, Wilcox test), Ace (*p* adj = 0.66, Wilcox test) (Fig. 1D). We did not observe any significant difference between the alpha diversity of the clean and natural samples using any of the above metrics. Overall, the results demonstrate that skin bacteria are integral part of the BM microbiome, rather than contaminants.

### The composition and diversity of human milk microbiome differs across the stages of lactation

**Fig. 2:**
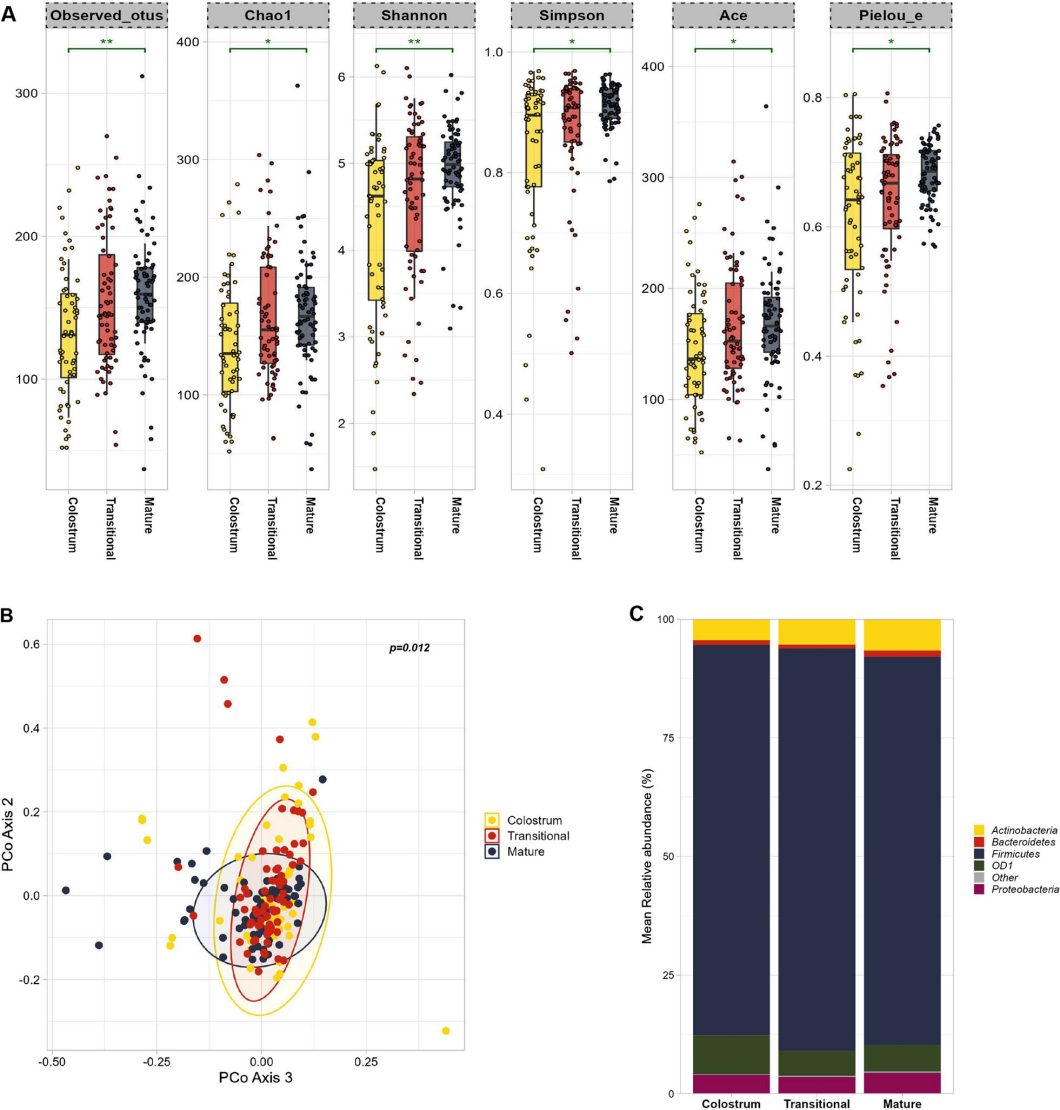
Comparison of the microbiota composition and diversity at different stages of lactation in BM samples. (**A**) Boxplots of Alpha-diversity indices: Observed OTUs; Chao1; Shannon; Simpson; Ace; Pielou_e. Boxes represent the interquartile range (IQR) between the first and third quartiles (25th and 75th percentiles, respectively), and the horizontal line inside the box defines the median. Whiskers represent the lowest and highest values within 1.5 times the IQR from the first and third quartiles, respectively. Statistical significance was identified by the Kruskal wallis test with false discovery rate (FDR)-Benjamini-Hochberg (BH) corrected *p* values; ns = non-significant; *p < 0.05; **p < 0.01; ***P < 0.001; yellow: colostrum, red: transitional, royal blue: mature BM samples (**B**) PCoA plots showing the beta diversity measure using weighted unifrac distances (*p* = 0.012); *p* values determined by ADONIS; yellow: colostrum, red: transitional, royal blue: mature BM samples. (**C**) Relative abundances of top five most abundant bacterial phylum in the three groups. The figure was generated using (RStudio v 2022.2.3.492 with R v 4.0.5)

BM dynamically adjusts to fulfill the immediate requirements of the infant, progressing through three distinct phases: colostrum, transitional milk, and mature milk. Throughout these lactation stages, both nutritional and non-nutritional constituents of BM exhibit variations [4]. In this context, our aim was to investigate whether a similar phenomenon is observed within the BM microbiome. Our overall analysis revealed notable variations in microbial diversity and richness across the colostrum, transitional, and mature milk stages (Fig. 2A). Colostrum has the lowest diversity, which progressively escalates as the milk matures, observed OTUs (*p* adj = 0.006, Kruskal–Wallis test), Shannon’s index (*p* adj = 0.006, Kruskal–Wallis test), Pielou’s evenness (*p* adj = 0.014, Kruskal–Wallis test), Simpson’s index (*p* adj = 0.011, Kruskal–Wallis test), Chao1 (*p* adj = 0.014, Kruskal–Wallis test), Ace (*p* adj = 0.014, Kruskal–Wallis test) (Fig. 2A). To determine differences in beta diversity according to lactation stage, PCoA plots were constructed based on the weighted unifrac (Fig. 2B) and Jaccard distance matrices (Additional file 1: Fig. S3A). Adonis variance analysis on both the matrices showed significant differences between the lactation stages (Adonis: *p* = 0.012 and *p* = 0.001 respectively) (Fig. 2B, Additional file 1: Fig. S3A).

**Fig. 3:**
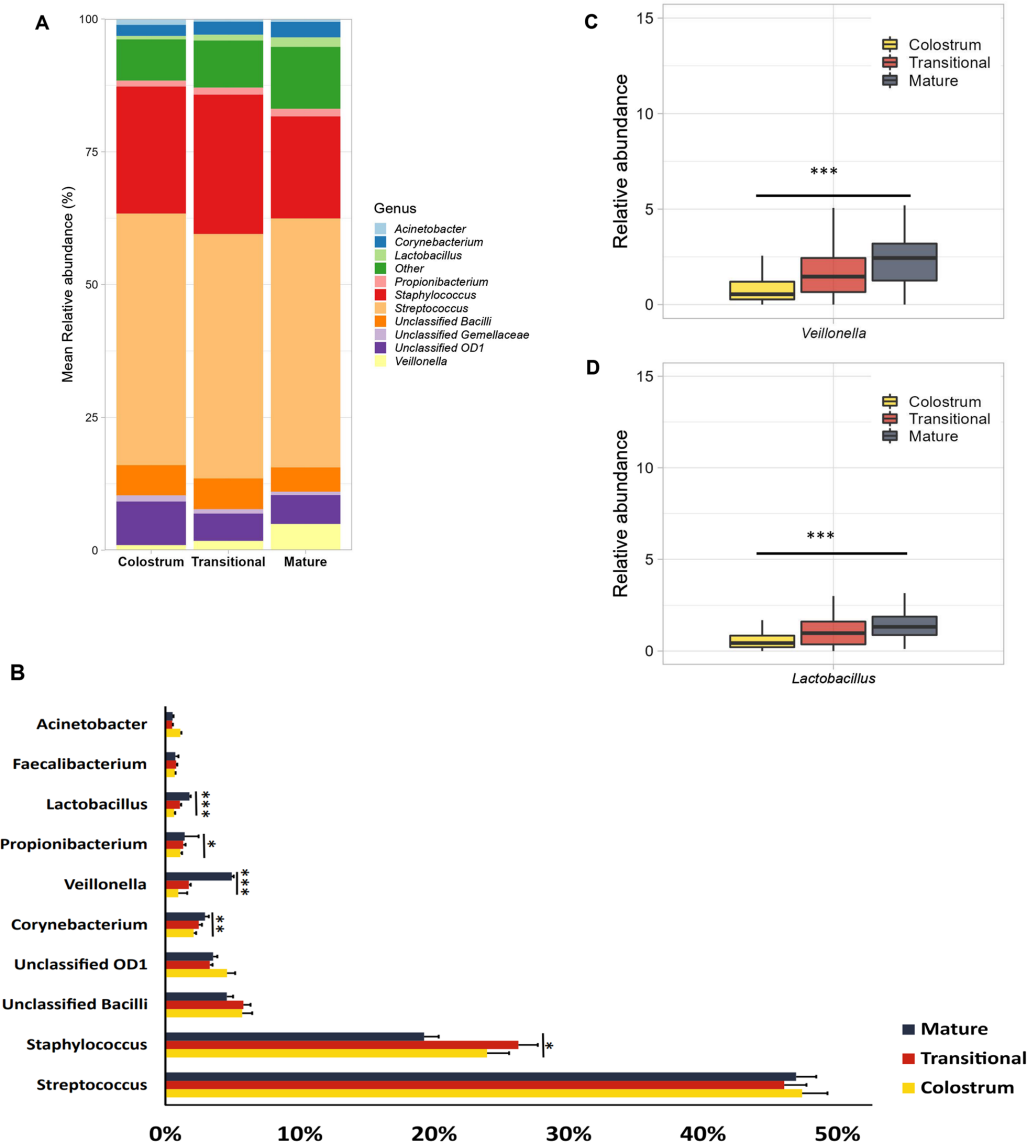
Comparison of the mean relative abundances of top genera identified in the three stages of lactation. (**A**) Stacked bar plots. Individual relative abundance box plots (**B**) *Veinollella* (**C**) *Lactobacillus* (**D**) Individual bar plots for the top 10 genus across the three stages of lactation. Statistical significance was identified by the Kruskal wallis test with false discovery rate (FDR)-Benjamini-Hochberg (BH) corrected *p *values; ns = non-significant; *p < 0.05; **p < 0.01; ***p < 0.001; ****p < 0.0001, yellow: colostrum, red: transitional, royal blue: mature BM samples. The figure was generated using (RStudio v 2022.2.3.492 with R v 4.0.5)

Several consistent phyla were identified across the lactation stages. Firmicutes, the most dominant phylum, exhibited dominance during early and mid-lactation, with its prevalence decreasing in mature BM samples (Kruskal–wallis, *p* < 0.05) (Fig. 2C, Additional file 1: Fig. S3B). Actinobacteria increased as the lactation progressed (Kruskal–Wallis test, *p* < 0.001) similarly the abundances of Bacteroidetes and Proteobacteria were both highest in mature milk samples (Kruskal–wallis test, *p* < 0.001, *p* = 0.02, respectively) (Fig. 2C, Additional file 1: Fig. S3B). At the genus level, *Streptococcus* was the most abundant genus (Fig. 3A). Moreover, during the transitional stage of milk, we observed that the abundance of *Staphylococcus* was the highest, as the milk matured milk*, Veillonella, Lactobacillus*, skin commensals *Corynebacterium* and *Propionibacterium* exhibited an significant upward trend in their relative abundances when compared to colostrum and transitional milk (Kruskal–wallis test, p < 0.05, respectively) (Fig. 3 A/B/C/D and Additional file 1: Fig. S4A/B). The 10 most abundant species across the lactation stages are represented in (Additional file 1: Fig. S5). Several species include lactic acid bacteria (i.e., *Lactobacillus iners*, *Unclassified Lactobacillus*), gut commensals (i.e., *Prevotella melaninogenica, Prevotella copri, Unclassified_Lachnospiraceae, Unclassified_Clostridiales*), oral commensals (*unclassified Veillonella, Rothia mucilaginosa*) as well as some environmental commensals found in soil, plant roots or water such as (*Burkholderia gladioli*) were found to be significantly different across the three lactation stages, in total 12 species found to be significantly different (Kruskal–Wallis *p.adj* < 0.5) are shown in (Additional file 2: Table S1).

### Preterm BM is compositionally distinct and high in species richness compared to term

**Fig. 4:**
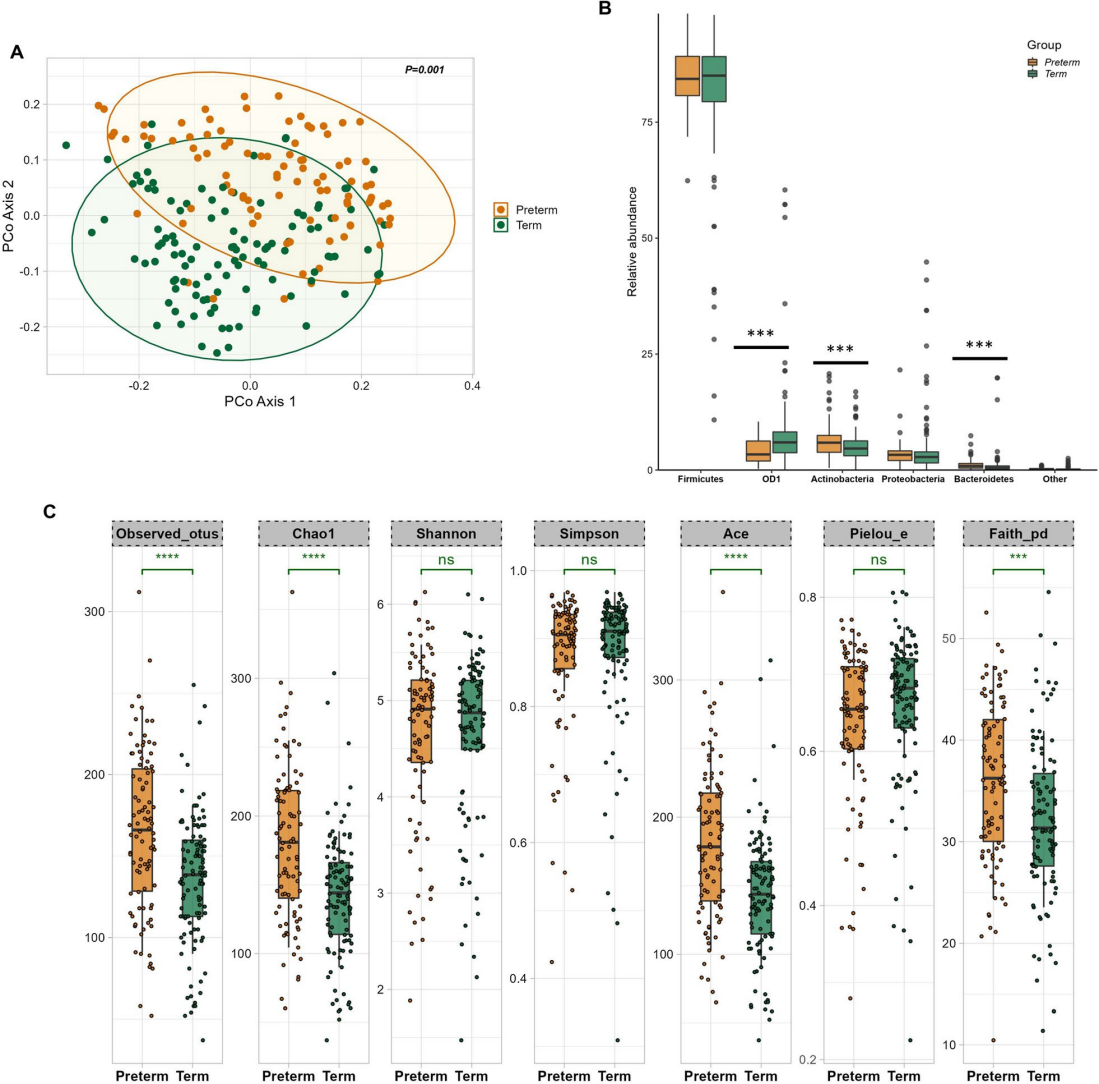
Comparison of the microbiota composition and diversity in preterm and term BM samples. (**A**) PCoA plots showing the beta diversity measure using unweighted unifrac distances (*p* = 0.001); *p* values determined by ADONIS; Preterm: brown, Term: green. (**B**) Mean relative abundances of top five abundant bacterial phyla in the two groups (**C**) Boxplots of Alpha-diversity indices: Observed OTUs; Chao1; Shannon; Simpson; Ace; Pielou_e; Faith_pd. Boxes represent the interquartile range (IQR) between the first and third quartiles (25th and 75th percentiles, respectively), and the horizontal line inside the box defines the median. Whiskers represent the lowest and highest values within 1.5 times the IQR from the first and third quartiles, respectively. Statistical significance was identified by the Wilcoxon test with false discovery rate (FDR)-Benjamini-Hochberg (BH) corrected *p* values. ns = non-significant; *p < 0.05; **p < 0.01; ***p < 0.001; ****p < 0.0001, brown: preterm, green: term. The figure was generated using (RStudio v 2022.2.3.492 with R v 4.0.5)

The maternal physiological state as well as the clinical characteristics of the child at birth, including gestational age, could potentially exert an influence on the composition of the BM microbiome. To assess this, we generated PCoA plots using both the Unweighted Unifrac and Bray–Curtis distance matrices. Adonis variance analysis applied to both matrices yielded results indicating significant compositional dissimilarity between preterm and term samples (*p* values = 0.001 respectively) (Fig. 4A and Additional file 1: Fig. S6A). Richness, which signifies the total number of species within a community, and evenness, indicating the equitable dispersion of species within that community, both constitute integral components of biodiversity. In our study, we employed several alpha diversity matrices encompassing Observed, Chao1, Ace (all assessing species richness), as well as Shannon, Simpson, and Pielou’s E (evaluating both richness and evenness). Among the richness matrices, namely observed OTUs (*p* adj = 0.000038, Wilcoxon test), Chao1 (*p* adj = 0.0000035, Wilcoxon test), Ace (*p* adj = 0.0000035, Wilcoxon test) and Faiths_pd incorporating phylogenetic distances in diversity calculations (*p* adj = 0.00073, Wilcoxon test) findings consistently indicated that preterm BM samples exhibited greater richness and diversity in comparison to term BM. Conversely, other matrices incorporating evenness, such as Pielou’s evenness (*p* adj = 0.1, Wilcoxon test), Shannon’s index (*p* adj = 0.8, Wilcoxon test), and Simpson’s index (*p* adj = 0.71, Wilcoxon test), showed non-significant disparities between the two sample groups (illustrated in Fig. 4C). The trends in alpha diversity observed in preterm and term BM samples remained consistent across various lactation stages, however after adjusting for p values significant differences were observed only in the preterm BM samples (Additional file 1: Fig. S7).

**Fig. 5:**
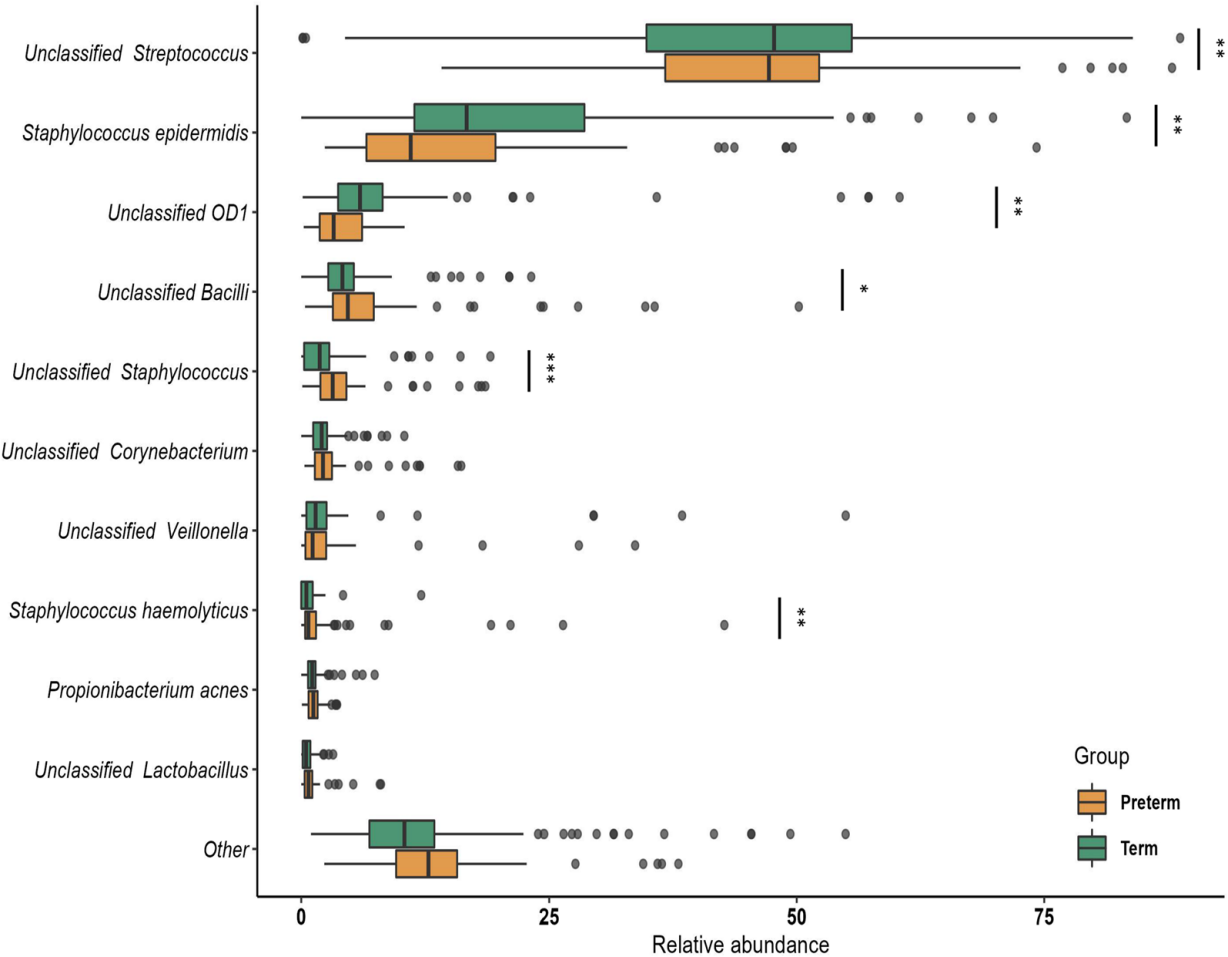
Comparison of relative abundance of top 10 species between the preterm and term BM samples. Boxes represent the interquartile range (IQR) between the first and third quartiles (25th and 75th percentiles, respectively), and the horizontal line inside the box defines the median. Whiskers represent the lowest and highest values within 1.5 times the IQR from the first and third quartiles, respectively. Statistical significance was identified by the Wilcoxon test with false discovery rate (FDR)-Benjamini-Hochberg (BH) corrected *p* values. ns = non-significant; *p < 0.05; **p < 0.01; ***p < 0.001; ****p < 0.0001, brown: preterm, green: term. The figure was generated using (RStudio v 2022.2.3.492 with R v 4.0.5)

From a taxonomic perspective, notable distinctions were evident in the phyla between the preterm and term groups. Actinobacteria and Bacteroidetes exhibited higher abundance in preterm BM samples (*p* adj < 0.0001, Wilcoxon test), while OD1 demonstrated greater prevalence in term samples (*p* adj < 0.0001, Wilcoxon test) (illustrated in Fig. 4B). The preterm samples were also enriched in common gut commensals such as *Faecalibacterium, Prevotella, Clostridium, Bacteroides, Enterobacter* (Additional file 1: Fig. S8), the significantly different genera between the term and preterm samples are detailed in Additional file 2: Table S2. Among the top 10 species *Staphylococcus haemolyticus, Propionibacterium acnes, Unclassified bacilli* were enriched in preterm BM samples. Whereas term samples were enriched in *Staphylococcus epidermidis, unclassified OD1,* and *unclassified Veillonella* among others (Fig. 5).

Overall, the results suggest that preterm birth significantly impacts the composition and diversity of BM microbiome.

The presence of antibiotics in a mother's system can have an impact on the composition of the BM microbiome. This impact can be attributed to several mechanisms, including alteration of maternal gut microbiota which can lead to an imbalance in the transmission of maternal gut bacteria to BM, influencing the milk's microbiome composition, or via a direct antibiotic transfer to BM. It is also worth noting that the type of antibiotics used, the time of administration during pregnancy, as well as the duration of treatment, can have varying effects on the BM microbiome. In our study, we did not find any significant impact of the exposure to antibiotics on the overall BM microbiota composition or diversity in samples collected during delivery (Additional file 1: Fig. S9A/B).

## Discussion

Traditionally, BM was believed to be sterile, however, recent research has shed light on its microbial diversity [6, 11, 12, 21, 42–50], revealing a potential influence on both the early gut colonization of the neonates [44] and the development of the immune system [42]. The origin of the BM microbiota remains a subject of ongoing and sometimes conflicting debate. Among the numerous hypotheses, the enteromammary and retrograde pathways are extensively discussed. The former suggests the transfer of maternal gut microbes to the mammary glands [9]. The enteromammary route requires transfer maternal/infant gut microbes to the mammary glands [51]. The evidence to support this concept is provided by the migration of B-lymphocytes from the maternal gut to the mammary gland, where they differentiate into plasmacytes and produce specific IgA antibodies to protect the infant from pathogens [52]. The retrograde pathway on the other hand involves transfer of infant oral microbiota during nursing or suckling, which in turn also leads to microbial colonization of the mammary ducts [53]. Other proposed sources for the bacteria in BM include maternal skin, oral, use of breast pump and its plausible that several pathways contribute to the microbial content of BM.

*Propionibacterium*, *Staphylococcus*, and *Corynebacterium* are few of the typical inhabitants of the adult skin [54] and are also found in BM [6, 11, 43, 55], presenting a possibility that maternal areolar skin microbiota may also contribute to the composition of BM microbiota. However, a comparison of the bacterial communities found on the sebaceous skin (like the ones found on breast) and those detected in the BM samples indicates that although the two communities share common taxa, major differences also exist [11, 56]. Among the universally predominant taxa in BM, *Staphylococcus* and *Streptococcus* are most frequent bacteria [57], they are also referred to as the core genera of BM microbiota [6]. Lackey et al., demonstrated that although the BM communities varied geographically, in samples collected from mothers across the USA, Spain, Ethiopia, Sweden, Gambia, Ghana, Kenya, and Peru, the BM core genera was universally composed of *Staphylococcus* and *Streptococcus* [6]. Consistent with the previous studies, our data also unveiled a similar pattern in out cohort of mothers. In order to eliminate the potential influence of skin-related microbial contamination in BM samples during the collection process; clean (breast was cleansed with povidone solution prior to sample collection) and natural (samples collected in their natural state without cleaning the breast) BM samples were collected. No appreciable differences in diversity or relative abundances were found when the bacterial communities from the two sample types were compared, suggesting that the bacterial communities present in our BM samples were not attributed to skin contamination; rather, they appear to be intrinsic constituents of the BM microbiota.

More than 800 different bacterial species, mainly from four major phyla *Firmicutes*, *Actinobacteria*, *Bacteroidetes*, and *Proteobacteria* have been reported in the BM samples [6, 11, 42, 43, 45–50, 56, 58, 59]. Among the top four phyla in our cohort, *Firmicutes* dominated with 83% of the overall composition, followed OD1 (6.33%), Actinobacteria (5.45%) and Proteobacteria (3.99%). OD1 has been reported in BM samples by other studies as a minor phyla [58], however, in our study it appeared as the second most abundant phylum. OD1, also known as *Parcubacteria*, is a group of uncultured bacteria discovered in various terrestrial water environments, lakes, and wetlands [60, 61]. These terrestrial and aquatics wetlands are common in both Thailand and Myanmar, and along the border area [62, 63]. This suggests that the composition of BM microbiota could be influenced by the surrounding environment. Additionally, we identified other soil and water-related bacteria in our BM cohort, including *Unclassified Pedobacter, Unclassified Planctomyces, Unclassified Rheinheimera, Burkholderia gladioli, Rhizobium, Micrococcus, Unclassified Rubrobacter, Rhodobacter, Bradyrhizobium*, *Novosphingobium*, *Pseudomonas*, *Sphingobium*, *Sphingopyxis*, *Sphingomonas* or *Xanthomonas*. This may indicate that leading a lifestyle in close contact with nature may possibly affect the enteromammary transmission of gut bacteria to the BM.

BM is divided into three distinct stages: colostrum, transitional milk, and mature milk, apparently adapting to the growing needs of the infant [4]. Few studies have tracked the progression of microbial communities in human milk over time [11, 64–66]. Cabrera-Rubio et al*.* were first to define the microbial communities in BM samples from 18 mothers collected at 2 days, 1 month and 6 months of lactation using pyrosequencing and qPCR [56]. They showed that BM undergoes considerable changes over time from colostrum to transitional and mature milk, including an increased abundance of typical oral occupant (e.g., *Veillonella*) in transitional and mature BM [56]. Consistent with the study, our data showed a progressive increase in oral bacteria *Veillonella* from colostrum to mature milk. This could be attributed to the increased interaction between BM and the infant's oral cavity as breastfeeding continues, potentially leading to the retrograde influence on the composition of BM's microbiota. A similar pattern emerged with other genera, such as *Lactobacillus*, *Corynebacterium*, *Propionibacterium* as their proportions increased when the milk matures. *Lactobacillus* have been reported to be more abundant in the gut of breast-fed neonates when compared with formula-fed babies [67]. Together with other probiotic bacteria *Lactobacillus*, have been shown to improve intestinal barrier functions in neonates by promoting mucosal barrier homeostasis, enhancing mucine production and reducing intestinal permeability [68] ultimately leading to a healthy immune system in early and adult life [69]. Additionally, *Lactobacillus, Propionibacterium*, and *Veillonella* are lactose fermenters that could prevent accumulation of lactate possibly neutralizing its unfavorable effects in infant gut [70–72], the above facts suggests that BM favors the colonization of the selective bacteria in the gut of the neonates.

Contrary to other studies [56, 66, 73], we observed an increase in diversity as lactation progresses, this phenomenon could potentially be attributed to the fluctuations in other biologically active constituents within milk throughout the breastfeeding period. Among these, Human Milk Oligosaccharides (HMOs), which function as metabolic substrates for specific intestinal microbes like *Lactobacillus* and *Bifidobacterium*, display varying concentrations across different stages of lactation [74]. The increase in BM diversity possibly contributes to the progression of the infant gut microbiota's maturation, considering that the diversity of the infant gut microbiota generally increases [75] in similar time intervals.

Previous studies have shown that prematurity impacts the other components of mother's milk: for instance protein content in preterm mother's milk is higher than in term mother's milk [76, 77]. Similarly concentration of amino acids, including valine, threonine and arginine is higher in preterm mother's milk [78]. Preterm BM appears also rich in sIgA but deficient in leptin [79–81]. *Streptococcus* was the predominant genera in our preterm BM samples, whereas the abundance of *Staphylococcus* was lower than previously reported [21]. In a case–control study examining the gut microbiota of 121 mothers with vaginal deliveries, the mothers giving birth prematurely were found to have lower abundance of *Streptococcus*, four days postpartum [82], whereas another study reported a higher abundance of *Streptococcus* in the gut microbiota of mothers who deliver preterm before delivery [83]. Evidence also suggests that PTB is associated with maternal Group B *Streptococcus* (GBS) colonization worldwide, previous work from SMRU suggests a low proportion (12%) of mothers carry Group B Streptococcus at birth [84, 85]. Due to limitation in analysis, we were not able to resolve the genus *Streptococcus* to species level, also we did not have the maternal gut microbiota samples from our cohort available for the present study. We also observed several gut commensals in our BM samples such as *Faecalibacterium, Prevotella, Clostridium, Bacteroides, Enterobacter* which could represent the “enteromammary” pathway of translocated maternal gut bacteria. Interestingly these commensals were significantly enriched in preterm BM samples as opposed to term samples which could provide them a competitive advantage in the colonization of the preterm infant gut. *Faecalibacterium*, *Prevotella*, *Clostridium* are major butyrate producers [86, 87] butyrate support enterocyte proliferation, increase barrier function via induction of tight junction proteins, also have a range of antimicrobial and anti-inflammatory effects [88] that could support the immature digestive and immune system of the preterm babies that have unique challenges at birth.

At species level, *Staphylococcus haemolyticus* was more abundant in the preterm BM samples whereas *Staphylococcus epidermidis* was enriched in the term BM samples. Previous studies have shown a high level of colonization of *Staphylococcus haemolyticus* in the gut and skin of preterm infants [89]. Whereas another study comparing bacterial diversity in the fecal samples of preterm and term infants showed lower levels of *Staphylococcus epidermidis* in the fecal samples of preterm infants [90]. This could provide indication of the vertical transmission of BM microbes from the mother to her infant, a process likely influenced by maternal health status. Preterm BM samples also demonstrated higher richness and diversity in terms of both core and rare taxa which could indicate an attempt to maximize ecosystem multifunctionality [91].

Antibiotic exposure is known to be associated with disruption in the richness, diversity and metabolic pathways of the intestinal microbiota [92]. Hence, it is conceivable that maternal antibiotic exposure may also perturb the BM microbiota. To reduce the risk of neonatal infections antibiotic treatment is often recommended in some cases [93]. Antibiotic exposure in utero and during infancy has been associated with an increased risk for the same diseases [94–96]. Recent studies have shown that intrapartum antibiotic exposure was significantly associated with changes in the milk microbial composition [97]. In our study, we did not find any significant impact of antibiotics exposure over the course of pregnancy or close to delivery neither on the diversity nor on the composition of the overall BM microbiota. These inconsistencies may have resulted from variations in the type, dosage, and timing of antibiotic administration, as well as from other environmental and genetic factors which require further investigation using larger cohorts and more studies.

Overall, we found significant differences in BM microbial communities depending on the lactation stage and gestational age. BM microbiota of PTB mothers was highly individualized likely suitable for the preterm infants. The strength of the study relies in the fact that we had a matching case control cohort which essentially minimizes biases and the effect of confounding factors. While results from this study are promising and warrant more research, it is worth noting that our study has few limitations. Firstly, low sequencing accuracy and low coverage of terminal regions associated with 16 S rRNA gene sequencing can result in low taxonomic resolution, as seen in our data where we had limited resolution at the species level. Secondly, the number of subjects that developed PTB was lower than the rates reported internationally, and a larger figure would have been more desirable for analytical purposes. Eventually, a deeper understanding of the determinants and progression of BM microbiota can provide insights into how the microbiota can be manipulated to improve infant health. These crucial early life phases and their effect on health and disease need to be deeply examined in order to support optimal microbial immune homeostasis.

### Supplementary Information

**Additional file 1: Figure S1.**Rarefaction curves of 16S rRNA gene sequence representing Observed OTUs in Clean and natural breast milk sample**s.** X -axis reports the number of sequences per samples. **Figure S2.** The relative abundance of bacteria in the breastmilk sample from the clean and natural groups at A) genus and B) species levels. **Figure S3.** A) Jaccard distance (*p* < 0.001); p values determined by ADONIS to compare different stages of lactation: colostrum (yellow), transitional (red), mature (royal blue) BM samples. B) The median relative abundance of bacteria in the breastmilk sample at different stages of lactation (colostrum, transitional and mature) BM samples at phylum and species levels. **Figure S4.** Differences in mean relative abundance of A) *Corynebacterium* B) *Propionibacterium* in the BM samples at three stages of lactation, (Colostrum, Transitional and Mature). **Figure S5.** Differences in mean relative abundance of top 10 species the BM samples across the three stages of lactation, Colostrum, Transitional and Mature BM sample in A) Individual bar plots B) Stacked bar plots. **Figure S6.** A) PCoA plot of Bray–Curtis distances (*p* = 0.001); *p* values determined by ADONIS to compare Preterm (brown) and Term (green) BM samples. B) The mean relative abundance of top 10 species in the preterm and term groups. **Figure S7.** A) Alpha diversity boxplots of Preterm (brown) and Term (green) BM samples across the 3 stages of lactation. **Figure S8.** Differences in mean relative abundance of A) *Prevotella* B) *Faecalibacterium C*) *Bacteroides D*) *Clostridium E*) Unclassified *Enterobacteriaceae* in the term and preterm BM sample. **Figure S9.** A) Alpha diversity boxplots of comparison of breastmilk samples relative to antibiotics exposure. Orange: Yes, and grey: no antibiotics B) Unweighted unifrac distances (*p* = 0.147 determined by ADONIS) to compare breastmilk samples relative to antibiotics exposure: yes (orange), no antibiotics (grey)

**Additional file 2: Table S1.** Statistical comparison table between stages of BM at the species level. **Table S2.** Statistical comparison table between the groups of BM samples at the genus level. Genus: Group – KRUSCAL-WALLIS.

## Data Availability

Available upon request.
