# Supplementary material for: Unveiling the dynamics of the breast milk microbiome: impact of lactation stage and gestational age
Source: J Transl Med. 2023 Nov 6;21:784. doi: 10.1186/s12967-023-04656-9 (PMC10629158; doi:10.1186/s12967-023-04656-9)
Supplement: Supplementary file 1 — Additional file 1: Figure S1.Rarefaction curves of 16S rRNA gene sequence representing Observed OTUs in Clean and natural breast milk samples. X -axis reports the number of sequences per samples. Figure S2. The relative abundance of bacteria in the breastmilk sample from the clean and natural groups at A) genus and B) species levels. Figure S3. A) Jaccard distance (p < 0.001); p values determined by ADONIS to compare different stages of lactation: colostrum (yellow), transitional (red), mature (royal blue) BM samples. B) The median relative abundance of bacteria in the breastmilk sample at different stages of lactation (colostrum, transitional and mature) BM samples at phylum and species levels. Figure S4. Differences in mean relative abundance of A) Corynebacterium B) Propionibacterium in the BM samples at three stages of lactation, (Colostrum, Transitional and Mature). Figure S5. Differences in mean relative abundance of top 10 species the BM samples across the three stages of lactation, Colostrum, Transitional and Mature BM sample in A) Individual bar plots B) Stacked bar plots. Figure S6. A) PCoA plot of Bray–Curtis distances (p = 0.001); p values determined by ADONIS to compare Preterm (brown) and Term (green) BM samples. B) The mean relative abundance of top 10 species in the preterm and term groups. Figure S7. A) Alpha diversity boxplots of Preterm (brown) and Term (green) BM samples across the 3 stages of lactation. Figure S8. Differences in mean relative abundance of A) Prevotella B) Faecalibacterium C) Bacteroides D) Clostridium E) Unclassified Enterobacteriaceae in the term and preterm BM sample. Figure S9. A) Alpha diversity boxplots of comparison of breastmilk samples relative to antibiotics exposure. Orange: Yes, and grey: no antibiotics B) Unweighted unifrac distances (p = 0.147 determined by ADONIS) to compare breastmilk samples relative to antibiotics exposure: yes (orange), no antibiotics (grey) [file 12967_2023_4656_MOESM1_ESM.pptx]

## Slide 1
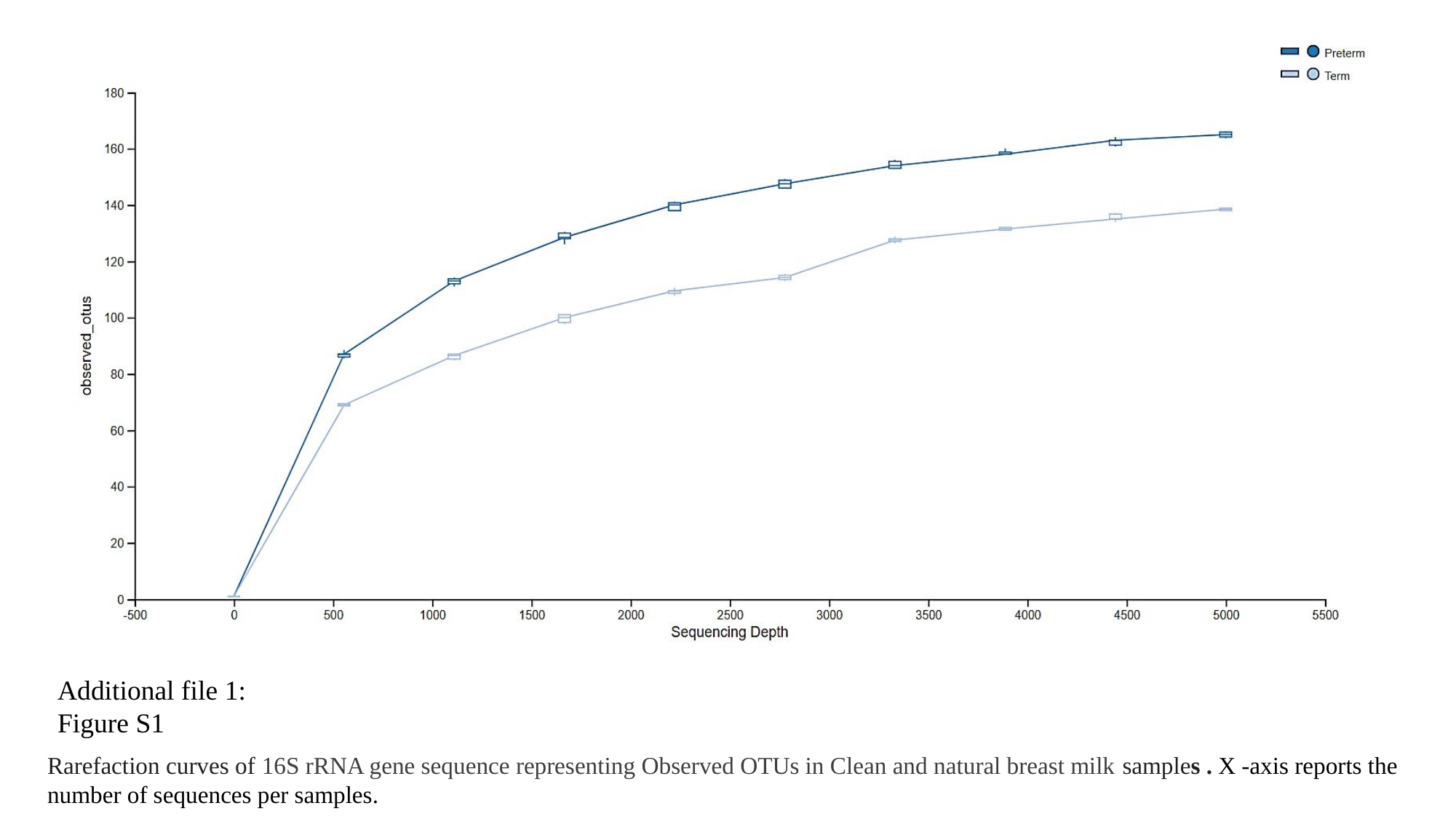

Additional file 1: Figure S1
Rarefaction curves of 16S rRNA gene sequence representing Observed OTUs in Clean and natural breast milk samples . X -axis reports the number of sequences per samples.

## Slide 2
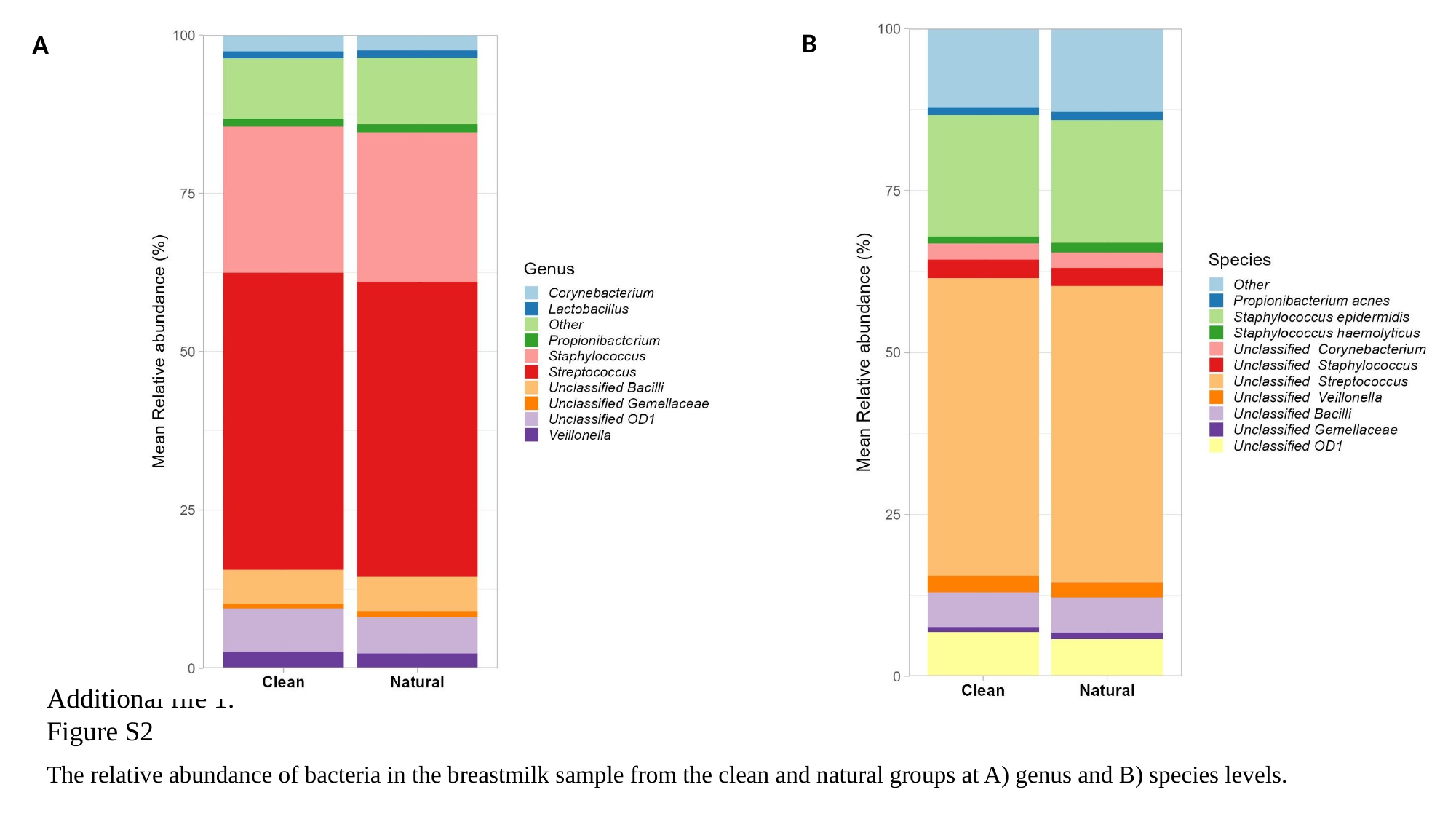

B
A
Additional file 1: Figure S2
The relative abundance of bacteria in the breastmilk sample from the clean and natural groups at A) genus and B) species levels.

## Slide 3
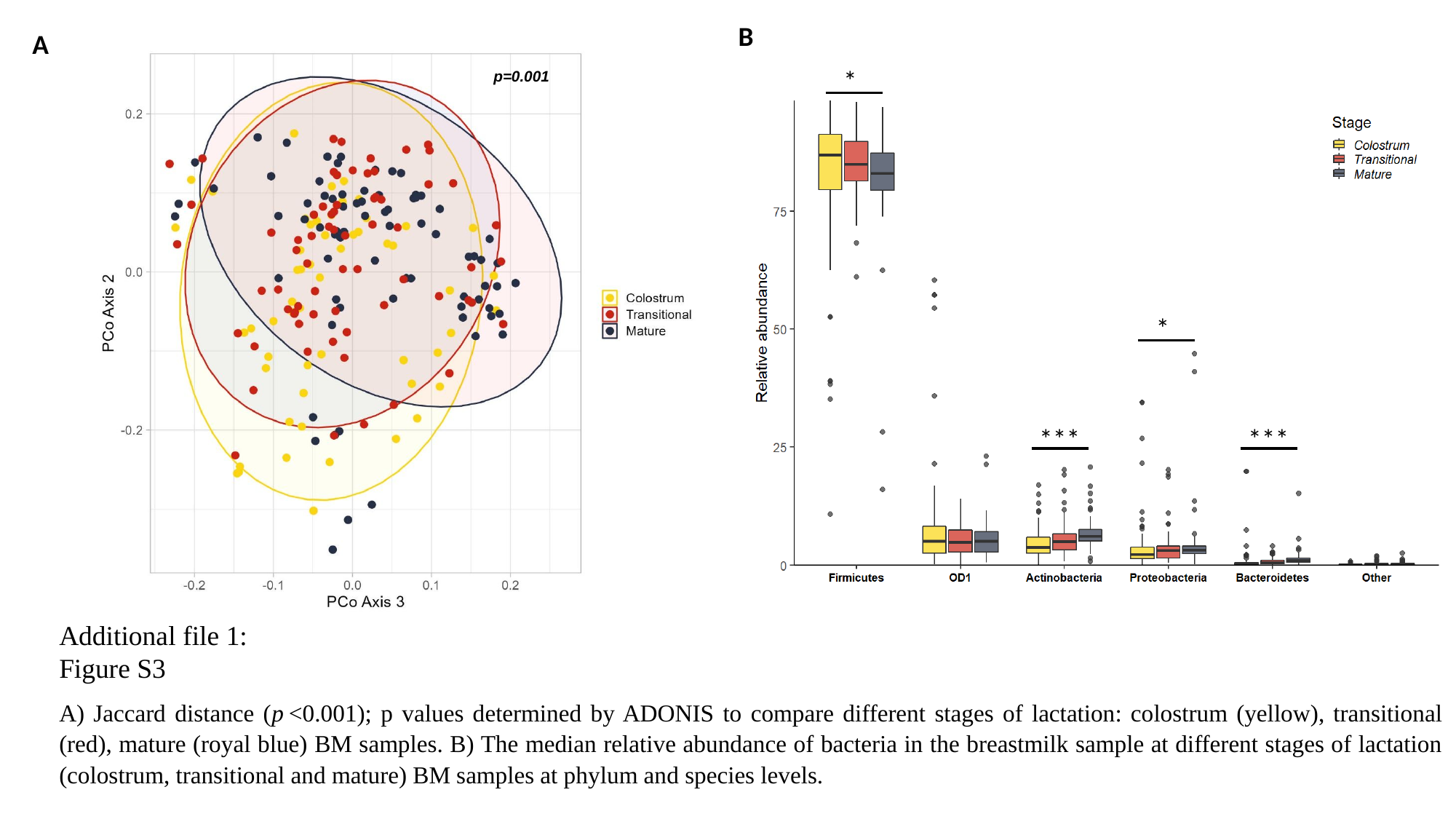

B
*
*
***
***
A
p=0.001
Additional file 1: Figure S3
A) Jaccard distance (p <0.001); p values determined by ADONIS to compare different stages of lactation: colostrum (yellow), transitional (red), mature (royal blue) BM samples. B) The median relative abundance of bacteria in the breastmilk sample at different stages of lactation (colostrum, transitional and mature) BM samples at phylum and species levels.

## Slide 4
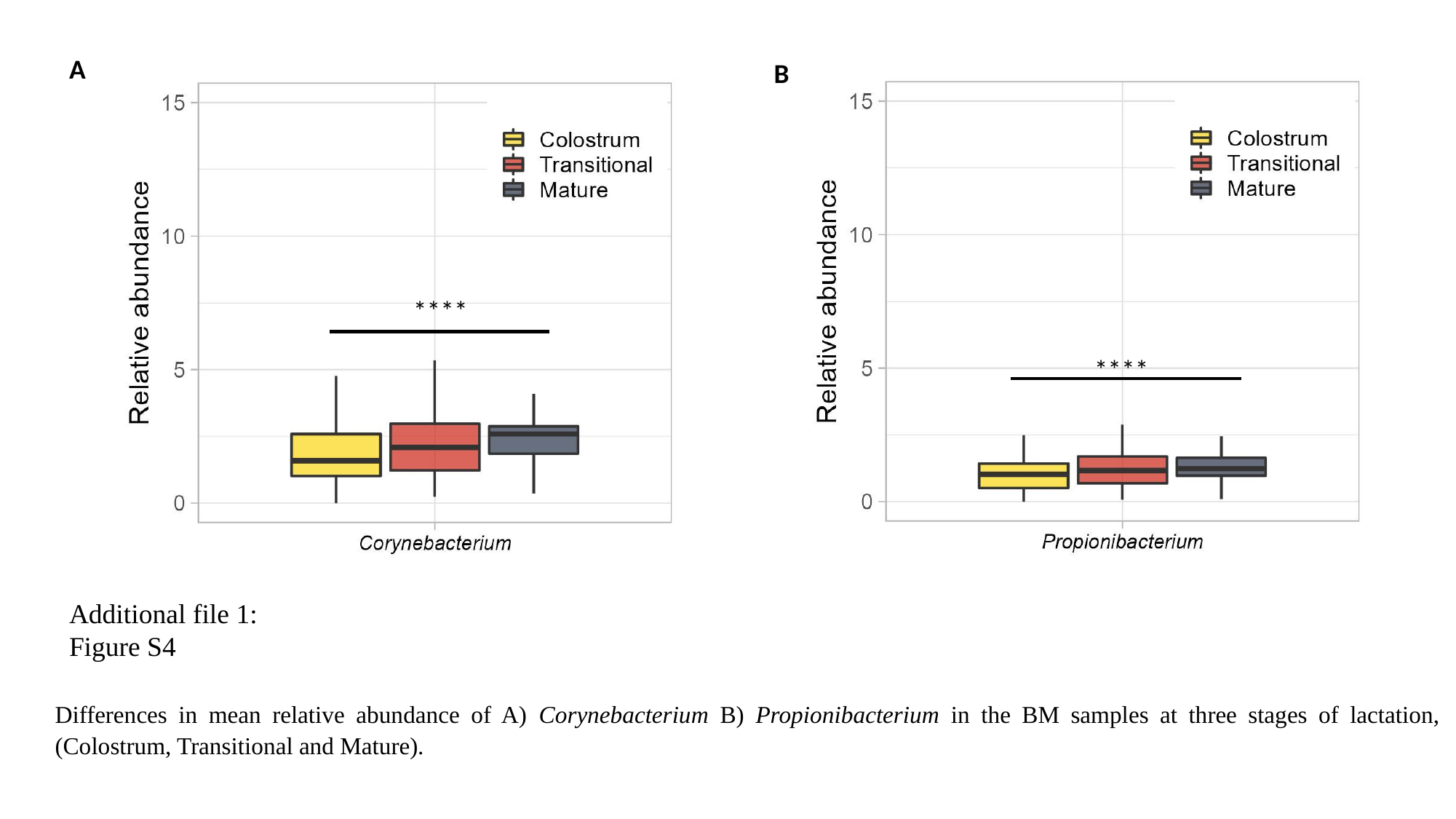

A
B
****
****
Additional file 1: Figure S4
Differences in mean relative abundance of A) Corynebacterium B) Propionibacterium in the BM samples at three stages of lactation, (Colostrum, Transitional and Mature).

## Slide 5
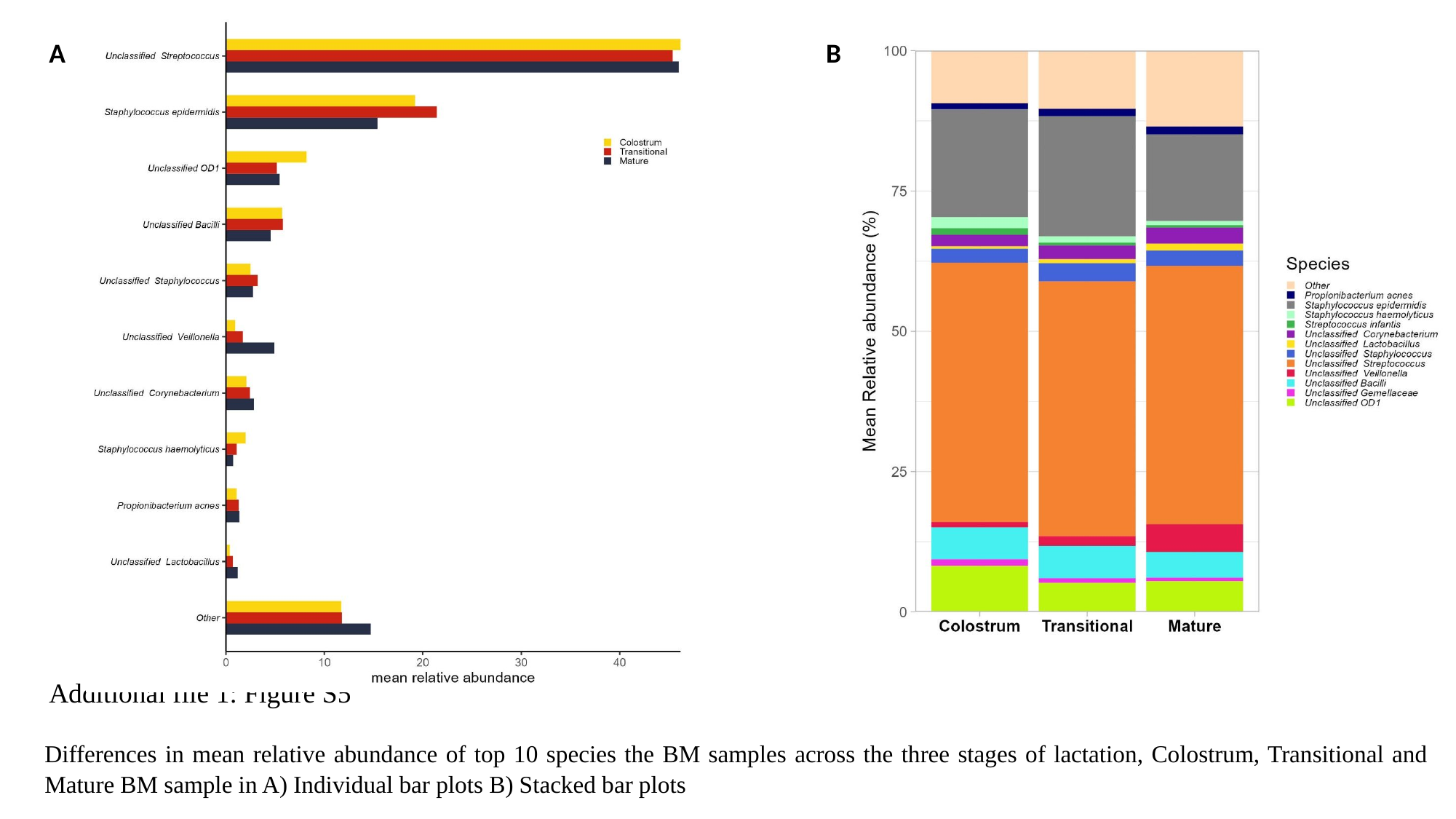

A
B
Additional file 1: Figure S5
Differences in mean relative abundance of top 10 species the BM samples across the three stages of lactation, Colostrum, Transitional and Mature BM sample in A) Individual bar plots B) Stacked bar plots

## Slide 6
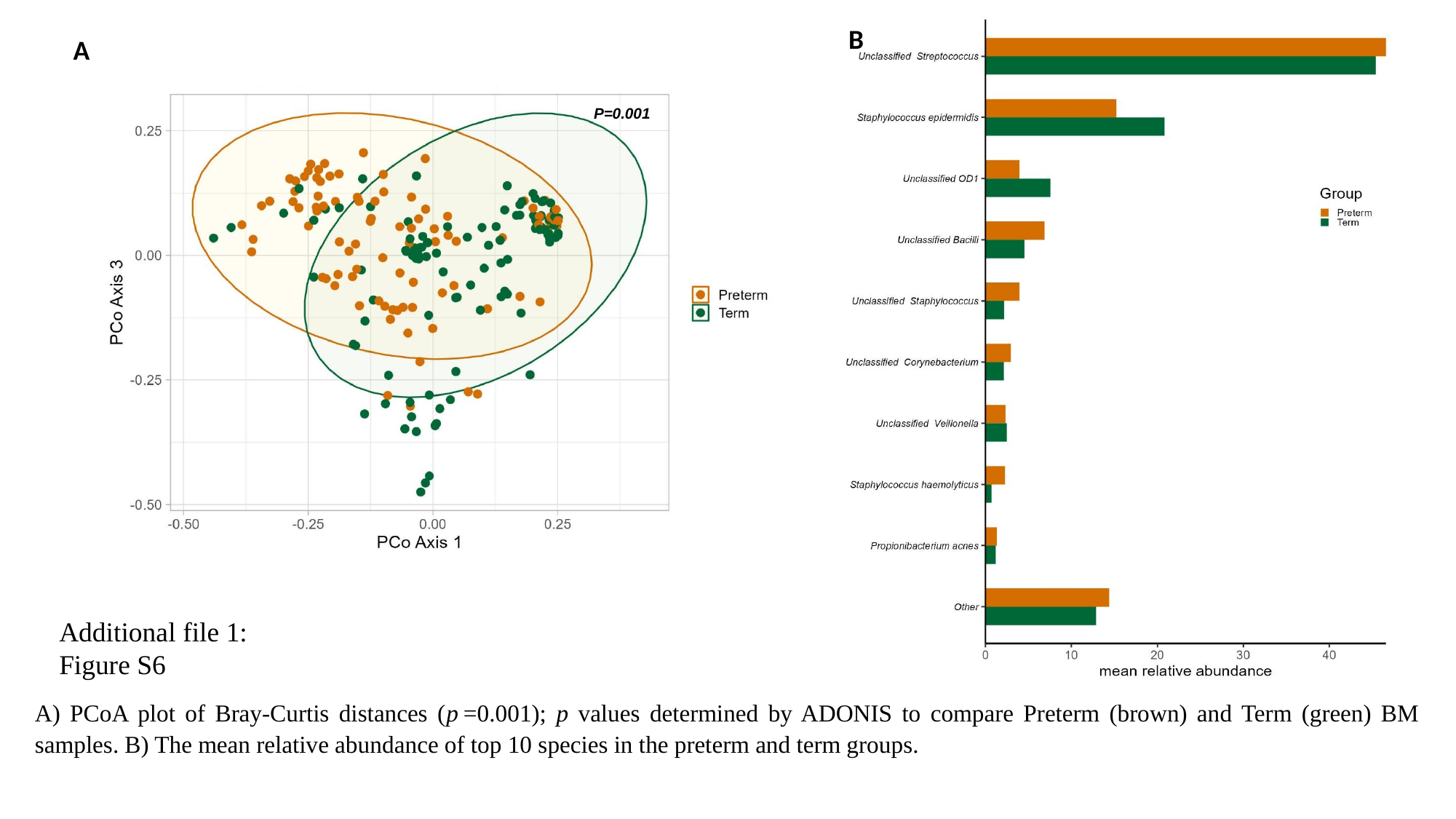

B
A
P=0.001
Additional file 1: Figure S6
A) PCoA plot of Bray-Curtis distances (p =0.001); p values determined by ADONIS to compare Preterm (brown) and Term (green) BM samples. B) The mean relative abundance of top 10 species in the preterm and term groups.

## Slide 7
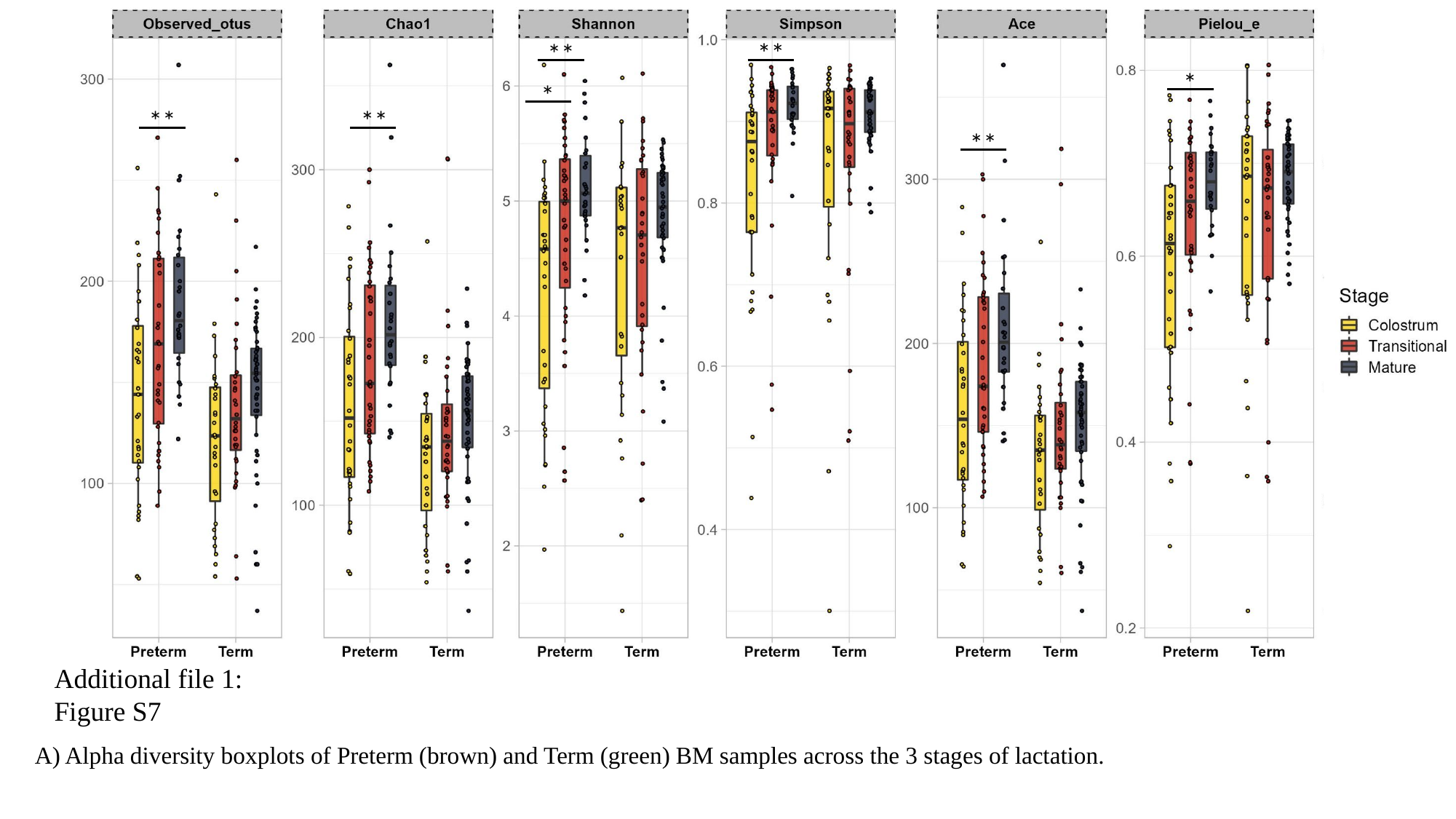

**
**
 *
 *
**
**
**
Additional file 1: Figure S7
A) Alpha diversity boxplots of Preterm (brown) and Term (green) BM samples across the 3 stages of lactation.

## Slide 8
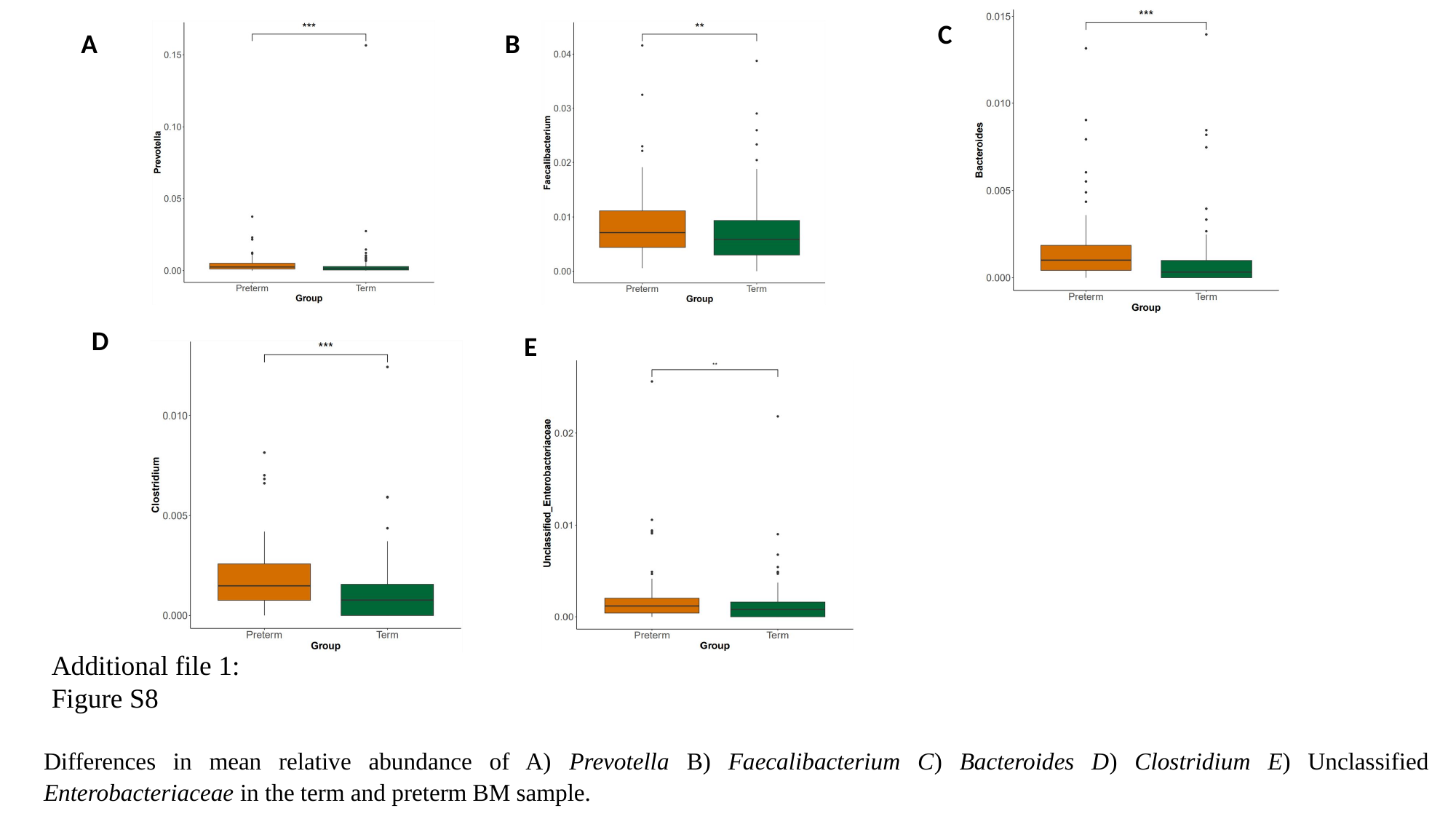

C
A
B
D
E
Additional file 1: Figure S8
Differences in mean relative abundance of A) Prevotella B) Faecalibacterium C) Bacteroides D) Clostridium E) Unclassified Enterobacteriaceae in the term and preterm BM sample.

## Slide 9
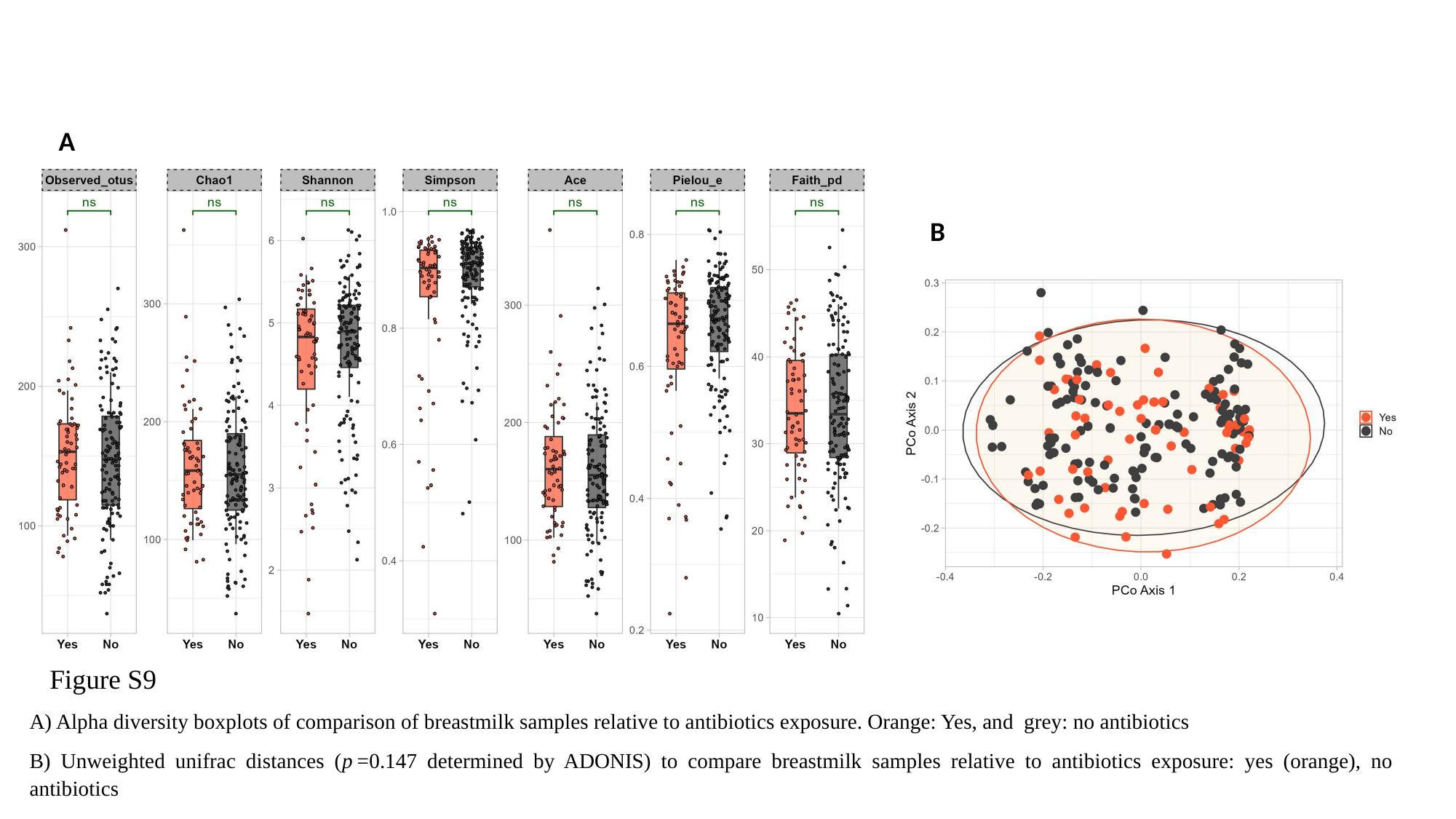

A
B
Additional file 1: Figure S9
A) Alpha diversity boxplots of comparison of breastmilk samples relative to antibiotics exposure. Orange: Yes, and grey: no antibiotics
B) Unweighted unifrac distances (p =0.147 determined by ADONIS) to compare breastmilk samples relative to antibiotics exposure: yes (orange), no antibiotics
 (grey)
