# Supplementary material for: Unveiling the dynamics of the breast milk microbiome: impact of lactation stage and gestational age
Source: J Transl Med. 2023 Nov 6;21:784. doi: 10.1186/s12967-023-04656-9 (PMC10629158; doi:10.1186/s12967-023-04656-9)
Supplement: Supplementary file 2 — Additional file 2: Table S1. Statistical comparison table between stages of BM at the species level. Table S2. Statistical comparison table between the groups of BM samples at the genus level. Genus: Group – KRUSCAL-WALLIS. [file 12967_2023_4656_MOESM2_ESM.docx]

Additional file 2: T**able S1: Species: Stage – KRUSCAL-WALLIS**

| Taxa | Group | P.adj | Significance |
| --- | --- | --- | --- |
| Unclassified_Veillonella | Mature | 8.0291E-11 | *** |
| Unclassified_Lactobacillus | Mature | 3.2086E-07 | *** |
| Staphylococcus_aureus | Mature | 0.00740609 | ** |
| Burkholderia_gladioli | Mature | 0.00389476 | ** |
| Rothia_mucilaginosa | Mature | 0.00413336 | ** |
| Unclassified_Enhydrobacter | Mature | 0.01310089 | * |
| Unclassified_Acinetobacter | Mature | 0.00413336 | ** |
| Unclassified_Lactobacillus_iners | Mature | 0.02984183 | * |
| Unclassified_Clostridiales | Mature | 9.6006E-05 | *** |
| Prevotella_copri | Mature | 0.02059468 | * |
| Unclassified_Lachnospiraceae | Mature | 0.00022046 | *** |
| Prevotella_melaninogenica | Mature | 1.4021E-07 | *** |
| Note: *- p<0.05, **- p<0.01, ***- p<0.001 | | | |

Additional file 2: Table **S2. Genus: Group – KRUSCAL-WALLIS**

| Taxa | Group | P.adj | Significance |
| --- | --- | --- | --- |
| Unclassified_Bacilli | Preterm | 0.02710823 | * |
| Unclassified_OD1 | Term | 0.005592105 | ** |
| Faecalibacterium | Preterm | 0.010914223 | * |
| Unclassified_Proteobacteria | Term | 0.005592105 | ** |
| Prevotella | Preterm | 0.001235729 | ** |
| Paracoccus | Preterm | 5.08432E-05 | *** |
| Kocuria | Preterm | 0.017959017 | * |
| Pseudomonas | Preterm | 0.009269516 | ** |
| Unclassified_Enterobacteriaceae | Preterm | 0.006113635 | ** |
| Clostridium | Preterm | 3.92846E-06 | *** |
| Bacteroides | Preterm | 9.04017E-07 | *** |
| Unclassified_Clostridiales | Preterm | 0.009269516 | ** |
| Unclassified_Lachnospiraceae | Preterm | 4.07807E-05 | *** |
| Note: *- p<0.05, **- p<0.01, ***- p<0.001 |  |  |  |
